# Supplementary material for: Hemolytic Activity and Cytotoxicity of Synthetic Nanoclays with Montmorillonite Structure for Medical Applications
Source: Nanomaterials (Basel). 2023 Apr 25;13(9):1470. doi: 10.3390/nano13091470 (PMC10180290; doi:10.3390/nano13091470)
Supplement: Supplementary file 1 [file nanomaterials-13-01470-s001.zip › nanomaterials-2295716-supplementary.pdf]

SUPPORTING INFORMATION

Hemolytic Activity and Cytotoxicity of Synthetic Nanoclays with Montmorillonite Structure for Medical Applications

Olga Yu. Golubeva *, Yulia A. Alikina, Elena Yu. Brazovskaya and Nadezhda M. Vasilenko

Laboratory of Silicate Sorbents Chemistry, Institute of Silicate Chemistry of Russian Academy of Sciences, Adm. Makarova emb., 2, 199034 St.Petersburg, Russia

* Correspondence: olga_isc@mail.ru

Summary

1	Description of the instruments and methods used	S2
2	X-ray diffraction study of the samples	S4
	Figure S1. Diffraction patterns of MT samples of different compositions	S5
3	References	S6

Description of the instruments and methods used

Chemical analysis of the samples for Si, Mg and Al content was performed gravimetrically using a quinolate of the silicon molybdenum complex and by complexometric titration. The sodium content of the studied samples was determined by atomic absorption spectroscopy (Thermo scientific iCE 3000, USA).

X-ray phase analysis of the samples was carried out using a powder diffractometer Rigaku Corporation, SmartLab 3 (CuKα - radiation, operating mode - 40 kV/40 mA; semiconductor point detector (0D) - linear (1D), θ-θ geometry, measurement range 2ϑ=5-70° (step 2θ=0.01°)).

The textural parameters of the materials were determined by the method of low-temperature adsorption-desorption of nitrogen. Isotherms were obtained using a Quantachrome NOVA 1200e instrument (Quantachrome Instruments, Boynton Beach, USA). Degassing was carried out at 300°C for 12 h. The specific surface area of the sample was calculated by the Brunauer–Emmett–Teller (BET) method using the NOVAWin (USA) software, based on the desorption curve.

The morphology of the samples was studied by scanning electron microscopy (SEM) by using a Carl Zeiss Merlin instrument (Oberkochen, Germany) with a field emission cathode. Powders of the samples were planted directly on conductive carbon tape without additional processing. Focused ion beam-scanning electron microscopy (FIB-SEM) was also used. A two-beam workstation with focused ion and scanning electron beams, a Carl Zeiss Auriga Laser with a field emission cathode, a column from GEMINI electronic optics, and an oil-free vacuum system were employed with a beam current range of 400 pA and acceleration voltage of 1.5–4 kV. Powders were placed directly on conductive carbon tape without additional processing.

The ζ-potential of the samples was determined using the particle size and zeta potential analyzer NaniBrook 90 PlusZeta (Brookehaven Instruments Corporation, USA). The electrokinetic (zeta) potential of the samples was determined using the particle size

and zeta potential analyzer NaniBrook 90 PlusZeta (Brookehaven Instruments Corporation, USA). The samples were a suspension obtained by dispersing 50 mg of sample in 20 ml of deionized water. Before measurements, the suspension was subjected to low power (50 W) ultrasonication for two minutes on an ultrasonic processor UP50H.

Cation exchange capacity (CEC) was measured by the ion-exchange reaction with hexammine cobalt (III) ions $[Co(NH_3)_6]^{3+}$ [1]. First, 500 mg of MT samples were dispersed in 30 ml of a 0.05 M hexammine cobalt (III) chloride solution. The dispersion was stirred for 2 h and centrifuged twice. The equilibrium concentrations of hexammine cobalt (III) ions were determined by the UV-visible absorbance of the supernatant solutions at the wavelength 473 nm (LEKI SS2109UV spectrophotometer, LEKI Instruments, Russia). CEC values were taken as the average of three measurements.

Measurement of the adsorption of benzene vapours was carried out on a vacuum adsorption unit using McBen-Bakr spring balances. Before measurements, the samples were degassed in vacuum with heating up to 200°C to a residual pressure in the system of $1.33 \cdot 10^{-3}$ Pa. The parameters of the microporous structure were determined from the data of benzene adsorption using the theory of micropore volume filling (TMVF) [2–4].

A hemolytic test was used to determine the ability of the studied samples to damage membranes of eukaryotic cells [5]. Human erythrocytes obtained from peripheral blood of healthy donors by a standard procedure [5,6] were used to determine the hemolytic activity. The studies were carried out according to the previously described method [7,8].

Human erythrocytes obtained from peripheral blood of healthy donors by a standard procedure were used to determine the hemolytic activity. The serial dilutions of the analyzed samples were prepared in a range of concentrations from 0.1 mg/mL to 10 mg/mL in 0.01 % acetic acid. 10 μL of each experimental sample was added to 90 μL of a 2.8 % suspension of erythrocytes. After adding the samples, 2.5 % concentration of the erythrocyte suspensions was achieved.

To obtain a positive control (100% erythrocyte lysis), 10 μL of Triton X-100 detergent diluted 10 times in an aqueous solution of 0.01 % acetic acid was added to 90 μL of the erythrocyte suspension. For negative control (0 % erythrocyte lysis) 10 μL of 0.01 % acetic acid was added to 90 μL of the erythrocyte suspension. Analyzed solutions (3 parallels

for each of the studied samples) were incubated at 37 °C for 30 min. The reaction was stopped by adding 150 μL of chilled phosphate buffer. Then the samples were centrifuged at 10,000 g for 4 min. The supernatant (200 μL) of each sample was taken and added to the wells of a 96-well plate (Costar, Corning Inc.). The optical density (OD_{540}) of the samples was measured using a SpectraMax 250 spectrophotometer (Molecular Devices, Sunnyvale, USA) at a wavelength of 540 nm to judge the content of hemoglobin released from destroyed erythrocytes.

The percentage of hemolysis was calculated using the following formula:

Hemolysis (%) = ((OD_{540}(sample) – OD_{540} (0% lysis)) / (OD_{540}(100% lysis) - OD_{540} (0% lysis)) x 100 %

The data are presented as means and standard deviations from the results obtained by three independent experiments, where the experimental and control samples were prepared in triplicates.

The result of the study is presented as a percentage of hemolysis corresponding to the content of hemoglobin released from destroyed erythrocytes after incubation of a suspension of erythrocytes with the studied samples of aluminosilicate.

The cytotoxic effect of the samples on eukaryotic cells was assessed using the MTT assay [9,10]. The experiments used an adhesive cell culture of human endothelial cells *Ea. hy 926*. The effect on cell survival of three concentrations (10, 5, 2.5 mg/ml) of samples was studied. 4 independent experiments were carried out, each of which had 2 parallels of experimental and control samples. Based on the data obtained, the cell survival was determined at various doses of the studied samples.

X-ray diffraction study of the samples

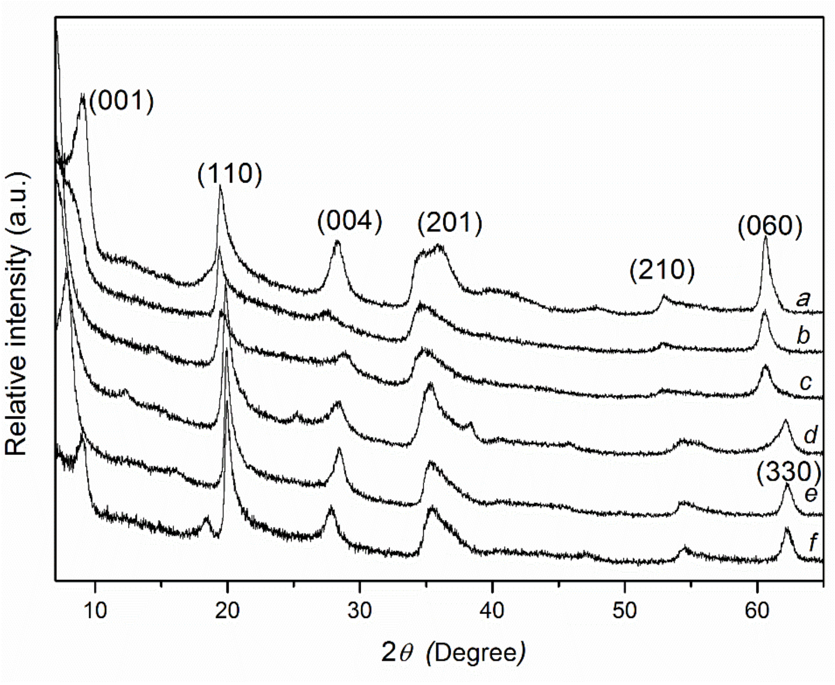

Figure S1. Diffraction patterns of MT samples of different compositions: (a) – Al0, (b) - Al0.2, (c) – Al05, (d) – Al1.2, (e) – Al1.8, (f) – Al1.9.

Diffraction patterns of MT samples (Fig. S1) show that the single-phase samples with MT structure were obtained, as evidenced by the position of the characteristic reflection peaks *hkl* at 7-9° (*001*), 19° (*110*), 28° (*004*), 35° (*201*), 60-62° ((*060*) and (*330*)). The nature of the diffraction patterns indicates a change in the structure of the samples with the increasing degree of substitution of magnesium atoms for aluminum and with the transition of the dioctahedral structure of the samples (2θ=60.8, d=1.48 Å, (*060*)) to trioctahedral (2θ=62.3, d=1.52 Å, (*330*)).

An average particle size was determined based on calculation according to the Scherrer formula at reflection band 2θ=19° (*110*), characterizing particle size in the plane perpendicular to axis *c*. The evaluation of the size of coherent scattering regions, conducted using the Scherrer formula at reflex d_{110}, showed that there is no dependence of the particle size in the plane perpendicular to the axis *c* on chemical composition. The particle size of all the samples is within 20±3 nm.

References

[1] F. Thomas, L.J. Michot, D. Vantelon, E. Montargès, B. Prélot, M. Cruchaudet, J.F. Delon, Layer charge and electrophoretic mobility of smectites, Colloids Surfaces A Physicochem. Eng. Asp. 159 (1999) 351–358. https://doi.org/https://doi.org/10.1016/S0927-7757(99)00291-5.

[2] M.M. Dubinin, Equivalent equations for micropore adsorption, Bull. Acad. Sci. USSR, Div. Chem. Sci. 27 (1978) 456–461. https://doi.org/10.1007/BF00923914.

[3] V. V Serpinski, T.S. Jakubov, Dubinin–Radushkevich Equation as the Equation for the Excess Adsorption Isotherm, Adsorpt. Sci. Technol. 10 (1993) 85–92. https://doi.org/10.1177/0263617499010001-408.

[4] B.P. Bering, M.M. Dubinin, V. V Serpinsky, Theory of volume filling for vapor adsorption, J. Colloid Interface Sci. 21 (1966) 378–393. https://doi.org/https://doi.org/10.1016/0095-8522(66)90004-3.

[5] Methods in Molecular Biology. Volume 78. Antibacterial Peptide Protocols Edited by William S. Shafer. The Humana Press, Totowa, NJ. 1997. x + 259 pp. 15.5 × 23.5 cm. ISBN 0-89603-408-9. $74.50., J. Med. Chem. 40 (1997) 4161. https://doi.org/10.1021/jm970565e.

[6] P.M. Kopeikin, M.S. Zharkova, A.A. Kolobov, M.P. Smirnova, M.S. Sukhareva, E.S. Umnyakova, V.N. Kokryakov, D.S. Orlov, B.L. Milman, S. V Balandin, P. V Panteleev, T. V Ovchinnikova, A.S. Komlev, A. Tossi, O. V Shamova, Caprine Bactenecins as Promising Tools for Developing New Antimicrobial and Antitumor Drugs., Front. Cell. Infect. Microbiol. 10 (2020) 552905. https://doi.org/10.3389/fcimb.2020.552905.

[7] O.Y. Golubeva, Y.A. Alikina, T. V Khamova, E. V Vladimirova, O. V Shamova, Aluminosilicate Nanosponges: Synthesis, Properties, and Application Prospects., Inorg. Chem. 60 (2021) 17008–17018. https://doi.org/10.1021/acs.inorgchem.1c02122.

[8] O.Y. Golubeva, Y.A. Alikina, T.A. Kalashnikova, Influence of hydrothermal synthesis conditions on the morphology and sorption properties of porous aluminosilicates with kaolinite and halloysite structures, Appl. Clay Sci. 199 (2020). https://doi.org/10.1016/j.clay.2020.105879.

[9] L. Tolosa, M.T. Donato, M.J. Gómez-Lechón, General Cytotoxicity Assessment by Means of the MTT Assay., Methods Mol. Biol. 1250 (2015) 333–348. https://doi.org/10.1007/978-1-4939-2074-7_26.

[10] M. Ghasemi, T. Turnbull, S. Sebastian, I. Kempson, The MTT Assay: Utility, Limitations, Pitfalls, and Interpretation in Bulk and Single-Cell Analysis., Int. J. Mol. Sci. 22 (2021). https://doi.org/10.3390/ijms222312827.
